# Supplementary material for: Identification of biomarkers associated with inflammatory response in Parkinson’s disease by bioinformatics and machine learning
Source: PLoS One. 2025 May 28;20(5):e0320257. doi: 10.1371/journal.pone.0320257 (PMC12118872; doi:10.1371/journal.pone.0320257)
Supplement: S1 File — (DOCX) [file pone.0320257.s002.docx]

# Data Availability Statement

The datasets generated and/or analyzed during the current study are available in the Gene Expression Omnibus (GEO) repository, under the accession number GSE20141、GSE20164 and GSE202210. The repository contains all relevant raw data needed to replicate the results of the study, including values for statistical analysis.
