# Supplementary material for: Identification of biomarkers associated with inflammatory response in Parkinson’s disease by bioinformatics and machine learning
Source: PLoS One. 2025 May 28;20(5):e0320257. doi: 10.1371/journal.pone.0320257 (PMC12118872; doi:10.1371/journal.pone.0320257)
Supplement: Attachment — Submitted filename: PlosOne_PD biomarker.docx [file pone.0320257.s003.docx]

Comments

The manuscript presents a promising approach to identifying biomarkers for Parkinson's Disease (PD) using bioinformatics and machine learning. The authors leverage gene expression data from the GEO database to find differentially expressed genes (DEGs) associated with the inflammatory response in PD. They then employ machine learning to identify candidate biomarker genes for further validation. Additionally, the use of single-cell transcriptomic analysis to explore candidate gene expression in different cell types strengthens the rationale. However, a few key areas require further clarification and improvement.

Specific Comments:

1. Figure Quality:

The quality of the figures needs significant improvement. The current resolution makes it difficult to evaluate the data and its relationship to the presented results. Ensure figures are clear, well-labeled, and have sufficient resolution for publication.

2. Validation Analysis:

The evaluation using the mRNA-miRNA network and drug sensitivity analysis requires a more elaborate discussion of the results. This includes:

A detailed explanation of the findings related to observed negative correlation with drugs. How negative correlation can help in finding the drug to putative drug targets.

Clarification on the rationale for considering weak correlations (Cor < 0.5) as validating evidence for drug sensitivity.

Discuss about relationship between the mRNA-miRNA network and putative pathways, whether the 17 miRNAs link to the discussed pathways.

3. Single-Cell Transcriptomic Analysis:

The discussion of the single-cell transcriptomic data needs further elaboration on the following aspects:

- Specificity of expression of the candidate biomarker genes across different cell types.
- Differential expression patterns observed in specific cell populations like neurons or glial cells.
- Potential drug targets identified through drug sensitivity analysis that could be effective for these cell types.

By addressing these concerns, the authors can strengthen the manuscript and enhance its clarity and impact.
