# Supplementary material for: Identification of biomarkers associated with inflammatory response in Parkinson’s disease by bioinformatics and machine learning
Source: PLoS One. 2025 May 28;20(5):e0320257. doi: 10.1371/journal.pone.0320257 (PMC12118872; doi:10.1371/journal.pone.0320257)
Supplement: Attachment — Submitted filename: Response to Reviewers.docx [file pone.0320257.s007.docx]

**Reviewer #1:**

1.Is the manuscript technically sound, and do the data support the conclusions?

Reviewer #1:yes

Response-1:Thank you very much for recognizing our research.

2.Has the statistical analysis been performed appropriately and rigorously?

Reviewer #1:yes

Response-2:Thank you very much for recognizing our research.

3.Have the authors made all data underlying the findings in their manuscript fully available?

Reviewer #1:yes

Response-3:Thank you very much for recognizing our research.

4.Is the manuscript presented in an intelligible fashion and written in standard English?

Reviewer #1:yes

Response-4:Thank you very much for recognizing our research.

5.The quality of the figures needs significant improvement. The current resolution makes it difficult to evaluate the data and its relationship to the presented results. Ensure figures are clear, well-labeled, and have sufficient resolution for publication.

Response-5:Thank you for your valuable comments. We attach great importance to the quality of the graphs and have re-exported the images in tif format at dpi=300 resolution as per the requirements of the journal.

6.Validation Analysis:
The evaluation using the mRNA-miRNA network and drug sensitivity analysis requires a more elaborate discussion of the results. This includes:
(1)A detailed explanation of the findings related to observed negative correlation with drugs. How negative correlation can help in finding the drug to putative drug targets.
(2)Clarification on the rationale for considering weak correlations (Cor < 0.5) as validating evidence for drug sensitivity.
(3)Discuss about relationship between the mRNA-miRNA network and putative pathways, whether the 17 miRNAs link to the discussed pathways.

Response-6:

Thank you for your valuable comments. Below is a detailed response to the above questions. In addition, we have added detailed descriptions to the manuscript in the appropriate places.

(1) Negative correlation helps to find potential drug targets of the drugs. The results showed that IL18R1 was negatively correlated with the drug BP-1-102 (Cor = -0.36, P = 0.004).RELA was negatively correlated with the drugs ARRY-162 (Cor = -0.37, P = 0.004) and Tamoxifen (Cor = -0.3, P = 0.018). A negative correlation means that when the expression level of the gene increases, the effect of the drug tends to diminish. Xu, F., et al. [1] found a negative correlation between camptothecin drugs and SLC1A5 expression in a drug sensitivity analysis of SLC1A5 expression with classical chemotherapeutic drugs.

(2) As this study is in the pre-exploratory stage, these weak correlations are still informative and may reveal potentially complex biological mechanisms. In addition, the relationship between genes and drug responses is usually nonlinear, so a single weak correlation does not imply a lack of biological significance. For example, Song, D., et al [2], in exploring the sensitivity of small molecule drugs associated with NRAV expression, found that the drug Sapitinib, which is weakly associated with NRAV expression, induced apoptosis and inhibited phosphorylated epidermal growth factor receptor and its downstream pathway.

(3) miRNAs are associated with the pathways discussed. In our study, we mainly focused on mRNA biomarkers, so the pathway analysis was performed based on mRNAs. Based on this, we mainly explored the relationship between mRNAs and their associated pathways and mined potential miRNA associations through mRNAs. However, based on your suggestion, we further explored the role of miRNA target genes in the relevant pathways (S1 Fig.tif). Through KEGG and GO analysis of miRNA target genes, we found that the pathways of miRNA target genes overlapped with the pathways we identified at the mRNA level, specifically the virus process and ECM-receptor interaction pathways. These pathways, although significant (P<0.05), were ranked low and therefore did not appear in the pathway heatmap for mRNAs. This result further supports the possible interaction of mRNA-miRNA networks in relevant biological processes.

**S1 Fig KEGG and GO analysis of miRNA target genes**

7. Single-Cell Transcriptomic Analysis:

The discussion of the single-cell transcriptomic data needs further elaboration on the following aspects:

(1)Specificity of expression of the candidate biomarker genes across different cell types.

(2)Differential expression patterns observed in specific cell populations like neurons or glial cells.

(3)Potential drug targets identified through drug sensitivity analysis that could be effective for these cell types.

By addressing these concerns, the authors can strengthen the manuscript and enhance its clarity and impact.

Response-7:

Thank you for your valuable comments. Below is a detailed description for the specific expression of IL18R1, NMUR1 and RELA in different cell types. In addition, we have also added a detailed description in the discussion section of Chapter 4 of the manuscript.

(1) IL18R1 is specifically expressed in T cells and Endothelial cells. It suggests that IL18R1 may play an important role in the immune response, especially in T cells. T cells are key cells of the immune system and are involved in recognizing and attacking infected or tumor cells[3]. Whereas IL18R1 is a subunit of the IL-18 receptor, IL-18 is a pro-inflammatory cytokine that is commonly associated with immune response and inflammation[4]. The specific expression of IL18R1 in T cells may be related to its role in the immune response[5]. RELA is expressed in almost all cell populations.RELA is part of the NF-κB family, and NF-κB plays an important role in many biological processes, including immune response, inflammatory response, cell survival and proliferation[6]. The expression of RELA in virtually all cell populations suggests that it is a fundamental and broadly regulated factor that may maintain essential functions and responsiveness in the physiological state of the cell, especially in response to inflammation, infection, or cellular damage[7]. NMUR1 is specifically expressed in T cells.NMUR1 is usually associated with neuropeptide signaling, which may influence the immune response by regulating T cell activation, proliferation, or function[8].

(2) In single-cell transcriptome analysis, IL18R1 and NMUR1 were found to have a small amount of expression in excitatory neurons (ExN), inhibitory neurons (InN) cells, but more often found to have a high specific expression in T cells and Endothelial cells. While RELA has high expression in excitatory neurons (ExN), inhibitory neurons (InN) cells.

(3) Thank you very much for your valuable questions. Our study focuses on screening biomarker genes by transcriptomics and verifying the specific expression of these markers in different cell types using single-cell transcriptomics, aiming to reveal their distribution and expression characteristics at the cellular level. Regarding drug sensitivity analysis, we have indeed performed preliminary correlation analysis at the level of transcriptomics data to identify potential drug targets. However, drug sensitivity analysis was not carried out on single-cell transcriptomics data as the main goal of this study was to validate the specific expression of the marker genes in different cell types rather than to explore the direct effects of the drugs on these cell types. However, in the future we will focus on drug sensitivity analysis on single-cell transcriptomics data.

**Reviewer #2:**

1.Is the manuscript technically sound, and do the data support the conclusions?

Reviewer #2:yes

Response-1: Thank you very much for recognizing our research.

2. Has the statistical analysis been performed appropriately and rigorously?

Reviewer #2:yes

Response-2: Thank you very much for recognizing our research.

3.Have the authors made all data underlying the findings in their manuscript fully available?

Reviewer #2:yes

Response-3: Thank you very much for recognizing our research.

4.Is the manuscript presented in an intelligible fashion and written in standard English?

Reviewer #2:yes

Response-4: Thank you very much for recognizing our research.

5.Introduction: PD has a set of well-defined features, such as bradykinesia with resting tremor, as well as nonmotor symptoms including autonomic dysfunction, incontinence, and cognitive impairme
*Changes need in Impairment

Response-5: Thank you for your valuable comments. We have amended the paragraph.

6.Fig 1. is not clear *
Image need in Higher definition

Response-6: Thank you for your valuable comments. We attach great importance to the quality of the graphs and have re-exported the images in tif format at dpi=300 resolution as per the requirements of the journal. And uploaded separately in the system.

7.Provided all images in low definition, looks blurred
*Change into High definition with resolution more than 300dpi, follow the author instruction for preparing the Figures

Response-7: Thank you for your valuable comments. We have re-exported the image to a resolution of 300 dpi as required by the journal and set the image format to tif and uploaded it separately in the system.

8.All images provided without figure legends
*Provide legends on the top or bottom of the Figures

Response-8: Thank you for your valuable comments. We have provided legends to the right or left of the images, for example, in Figure 2A, Parkinson's patients are abbreviated as PD and the corresponding legend is in blue. Except for that, images were uploaded individually as required by the journal. Figure captions for each figure are placed in the body of the manuscript.

9.The author not followed the author instructions
*No line numbers mentioned. Use continuous line numbers

Response-9: Thank you for your valuable comments. We have added line numbers and page numbers to the manuscript as required by the journal manuscript format.

10.The author not followed reference style of the journal
*Follow the author instructions and reformat the references

Response-10: Thank you for your comments. We have updated the reference format and changed it to the “Vancouver” style required by the journal.

11. Finally, the three biomarkers were found to be closely associated with excitatory neurons and microglia in single-cell transcriptomic analysis. In conclusion, IL18R1, NMUR1 and RELA could be potential therapeutic targets for PD in inflammatory response and new biomarkers for PD diagnosis.
    *if possible show the drug target or biomarker interactions. Also show how the new biomarkers reacts with genes and show the response on inflammation.

Response-11: Thank you for your valuable comments. Below is a detailed response to your questions.

(1) Biomarker interactions: we have mapped the correlation heatmap of differentially expressed genes for inflammatory response in Section 3.1 of the article, which includes the correlation of the biomarkers screened, IL18R1, NMUR1 and RELA.

(2) Since our focus is on exploring the interactions between biomarkers and drugs, the analysis of drug-drug interactions is not addressed. In Section 3.7, the interpretation of interactions between drug targets and biomarkers is carried out. The sensitivities of three biomarkers, IL18R1, NMUR1 and RELA, corresponding to different drugs were detailed, where IL18R1 was positively associated with the Alisertib (Cor = 0.31, P = 0.016), Azacitidine (Cor = 0.33, P = 0.01) and Motesanib (Cor = 0.35, P = 0.007) drugs, IL18R1 was positively correlated with BP-1-102 (Cor = -0.36, P = 0.004) drug, NMUR1 was positively correlated with Lexibulin (Cor = 0.31, P = 0.017) and norvir (Cor = 0.48, P < 0.001) drugs, and RELA was positively correlated with CAMPTOTHECIN (Cor = 0.32, P = 0.012) and Clofarbine (Cor = 0.3, P = 0.019) drugs, and RELA was positively correlated with ARRY-162 (Cor = -0.37, P = 0.004) and Tamoxifen (Cor = -0.3, P = 0.018) and 7 other drugs were negatively correlated.

(3) Biomarkers associated with inflammatory response Explanation: a. The experiments were first performed by searching for candidate genes that might serve as biomarkers among the genes associated with the inflammatory response. b. These biomarkers were found to be associated with the differential expression of Mast cells and Neutrophils cells by immune cell infiltration analysis. c. The biomarkers were found to be associated with the differential expression of Mast cells and Neutrophils cells by the immune cell infiltration analysis. Mast cells and Neutrophils cells are key cells involved in immune responses and inflammatory processes[9, 10]. Therefore, the differential expression of markers with them may imply that these markers play a role in regulating the inflammatory response.

# References

1.	Xu, F., H. Wang, H. Pei, Z. Zhang, L. Liu, L. Tang, S. Wang, and B.-C. Ren, *SLC1A5 prefers to play as an accomplice rather than an opponent in pancreatic adenocarcinoma.* Frontiers in Cell and Developmental Biology, 2022. **10**: p. 800925.

2.	Song, D., X. Wang, Y. Wang, W. Liang, J. Luo, J. Zheng, and K. Zhu, *Integrated analysis of N1-methyladenosine methylation regulators-related lncRNAs in hepatocellular carcinoma.* Cancers, 2023. **15**(6): p. 1800.

3.	Ruf, B., T.F. Greten, and F. Korangy, *Innate lymphoid cells and innate-like T cells in cancer—at the crossroads of innate and adaptive immunity.* Nature Reviews Cancer, 2023. **23**(6): p. 351-371.

4.	Di, Y., Z. Wang, J. Xiao, X. Zhang, L. Ye, X. Wen, J. Qin, L. Lu, X. Wang, and W. He, *ACSL6-activated IL-18R1–NF-κB promotes IL-18–mediated tumor immune evasion and tumor progression.* Science Advances, 2024. **10**(38): p. eadp0719.

5.	Lutz, V., V.M. Hellmund, F.S. Picard, H. Raifer, T. Ruckenbrod, M. Klein, T. Bopp, R. Savai, P. Duewell, and C.U. Keber, *IL18 receptor signaling regulates tumor-reactive CD8+ T-cell exhaustion via activation of the IL2/STAT5/mTOR pathway in a pancreatic cancer model.* Cancer immunology research, 2023. **11**(4): p. 421-434.

6.	Guo, Q., Y. Jin, X. Chen, X. Ye, X. Shen, M. Lin, C. Zeng, T. Zhou, and J. Zhang, *NF-κB in biology and targeted therapy: new insights and translational implications.* Signal Transduction and Targeted Therapy, 2024. **9**(1): p. 53.

7.	Khan, A., Y. Zhang, N. Ma, J. Shi, and Y. Hou, *NF-κB role on tumor proliferation, migration, invasion and immune escape.* Cancer Gene Therapy, 2024: p. 1-12.

8.	Ye, Y., Z. Liang, and L. Xue, *Neuromedin U: potential roles in immunity and inflammation.* Immunology, 2021. **162**(1): p. 17-29.

9.	Mencarelli, A., P. Bist, H.W. Choi, H.J. Khameneh, A. Mortellaro, and S.N. Abraham, *Anaphylactic degranulation by mast cells requires the mobilization of inflammasome components.* Nature Immunology, 2024. **25**(4): p. 693-702.

10.	Mihlan, M., S. Wissmann, A. Gavrilov, L. Kaltenbach, M. Britz, K. Franke, B. Hummel, A. Imle, R. Suzuki, and M. Stecher, *Neutrophil trapping and nexocytosis, mast cell-mediated processes for inflammatory signal relay.* Cell, 2024. **187**(19): p. 5316-5335. e28.
