# Supplementary material for: Identification of biomarkers associated with inflammatory response in Parkinson’s disease by bioinformatics and machine learning
Source: PLoS One. 2025 May 28;20(5):e0320257. doi: 10.1371/journal.pone.0320257 (PMC12118872; doi:10.1371/journal.pone.0320257)
Supplement: Attachment — Submitted filename: Untitled 1.docx [file pone.0320257.s006.docx]

Comments

I am pleased with the justifications and improvements made to the manuscript. However, I have noticed that some of the provided justifications have not been incorporated into the manuscript text. I recommend updating the discussion section to reflect these justifications for greater clarity and alignment with the revisions.
