# Supplementary material for: Identification of biomarkers associated with inflammatory response in Parkinson’s disease by bioinformatics and machine learning
Source: PLoS One. 2025 May 28;20(5):e0320257. doi: 10.1371/journal.pone.0320257 (PMC12118872; doi:10.1371/journal.pone.0320257)
Supplement: Attachment — Submitted filename: Response_to_Reviewers_auresp_2.docx [file pone.0320257.s009.docx]

**Reviewer #1:**

1. I am pleased with the justifications and improvements made to the manuscript. However, I have noticed that some of the provided justifications have not been incorporated into the manuscript text. I recommend updating the discussion section to reflect these justifications for greater clarity and alignment with the revisions.

Response-1: Thank you so much for your positive feedback and valuable suggestions on our manuscript. We have updated Chapter 4 (Discussion) and Chapter 5 (Conclusion) of the manuscript to ensure that all the explanations we provided in our responses are fully integrated into the main text. Additionally, in response to your question, "Discuss the relationship between the mRNA-miRNA network and the putative pathways, and whether the 17 miRNAs are related to the discussed pathways," we have provided a detailed description in Section 3.8.

**Reviewer #2:**

1.Again, the author given All the images in lo w resolution. Kindly provide with high definition as mentioned in Author instruction.

Response-1: Thank you for your attention to the issue of image resolution. We have uploaded the images in TIF format with a resolution of 300 dpi, as required by the journal. However, we have noticed that the submission system compresses the images when generating the PDF, which results in a reduction of image clarity. This compression is an automatic process that we cannot control. We assure you that the original images we uploaded meet the journal’s high-resolution requirements. You can download the original images separately through the submission system, or you can click on the links to the original images within the PDF to view them in high resolution.
