# Supplementary material for: Identification of biomarkers associated with inflammatory response in Parkinson’s disease by bioinformatics and machine learning
Source: PLoS One. 2025 May 28;20(5):e0320257. doi: 10.1371/journal.pone.0320257 (PMC12118872; doi:10.1371/journal.pone.0320257)
Supplement: Attachment — Submitted filename: CommentsR2.docx [file pone.0320257.s008.docx]

Comments

I went through the revised manuscript submitted by the author. The manuscript is in now acceptable format.
