# Supplementary material for: Identification of biomarkers associated with inflammatory response in Parkinson’s disease by bioinformatics and machine learning
Source: PLoS One. 2025 May 28;20(5):e0320257. doi: 10.1371/journal.pone.0320257 (PMC12118872; doi:10.1371/journal.pone.0320257)
Supplement: Attachment — Submitted filename: Reviewer Comments.docx [file pone.0320257.s004.docx]

**Reviewer Comments**

| **No.** | **Author submission** | **Changes needed** |
| --- | --- | --- |
| 1 | **Introduction:** PD has a set of well-defined features, such as bradykinesia with resting tremor, as well as nonmotor symptoms including autonomic dysfunction, incontinence, and cognitive impairme[4]. | Changes need in Impairment |
| 2 | Fig 1. is not clear | Image need in Higher definition |
| 3 | Provided all images in low definition, looks blurred | Change into High definition with resolution more than 300dpi, follow the author instruction for preparing the Figures |
| 4 | All images provided without figure legends | Provide legends on the top or bottom of the Figures |
| 5 | The author not followed the author instructions | No line numbers mentioned. Use continuous line numbers |
| 6 | The author not followed reference style of the journal | Follow the author instructions and reformat the references |
| 7 | Finally, the three biomarkers were found to be closely associated with excitatory neurons and microglia in single-cell transcriptomic analysis. In conclusion, IL18R1, NMUR1 and RELA could be potential therapeutic targets for PD in inflammatory response and new biomarkers for PD diagnosis. | if possible show the drug target or biomarker interactions. Also show how the new biomarkers reacts with genes and show the response on inflammation. |
